# Determinants of treatment-seeking behavior during self-reported febrile illness episodes using the socio-ecological model in Kilombero District, Tanzania

**DOI:** 10.1186/s12889-021-11027-w

**Published:** 2021-06-05

**Authors:** Caroline M. Mburu, Salome A. Bukachi, Khamati Shilabukha, Kathrin H. Tokpa, Mangi Ezekiel, Gilbert Fokou, Bassirou Bonfoh, Rudovick Kazwala

**Affiliations:** 1 Institute of Anthropology, Gender and African Studies, University of Nairobi, Nairobi, Kenya; 2 Centre Suisse de Recherches Scientifiques en Côte d’Ivoire, Abidjan, Côte d’Ivoire; 3 Muhimbili University of Health and Allied Sciences, Dar es Salaam, Tanzania; 4 Sokoine University of Agriculture, Morogoro, Tanzania

**Keywords:** Febrile illness, Treatment-seeking behavior, Agro pastoralists, Socio-ecological model

## Abstract

**Background:**

Febrile diseases in Sub-Saharan Africa cause acute and chronic illness. Co-infections are common and these diseases have a complex etiology that includes zoonoses. For the implementation of appropriate treatment and control strategies, determinants of lay treatment-seeking behavior by the affected communities need to be understood. The objective of this study was to explore, using the socio-ecological model, the determinants of treatment-seeking actions among self-identified febrile illness cases in the Kilombero District of Tanzania.

**Methods:**

Thirty-nine in-depth interviews were conducted with 28 men and 11 women in three villages in Kilombero district. These villages were purposively selected due to malaria endemicity in the area, animal husbandry practices, and proximity to livestock-wildlife interaction, all risk factors for contracting febrile zoonotic infections. Thematic analysis was conducted on the interviews to identify the key determinants of treatment-seeking actions.

**Results:**

Study participants attributed febrile illnesses to malaria, typhoid and urinary tract infections. Treatment-seeking behavior was an iterative process, influenced by individual, socio-cultural, ecological and policy factors. Age, expendable income, previous history with a febrile illness, perceptions on disease severity, seasonal livelihood activities and access to timely healthcare were some of the determinants. Self-treatment with pharmaceutical drugs and herbs was usually the initial course of action. Formal healthcare was sought only when self-treatment failed and traditional healers were consulted after the perceived failure of conventional treatment. Delays in seeking appropriate health care and the consultation of medically unqualified individuals was very common.

**Conclusion:**

The results imply that treatment-seeking behavior is shaped by multiple factors across all levels of the socio-ecological model. Public policy efforts need to focus on facilitating prompt health care seeking through community education on the complicated etiology of febrile illnesses. Improved access to timely treatment and better differential diagnostics by health professionals are essential to ensure correct and appropriate treatment and to reduce reliance of patients on unqualified persons.

**Supplementary Information:**

The online version contains supplementary material available at 10.1186/s12889-021-11027-w.

## Background

In Sub-Saharan Africa, diseases causing fever, contribute significantly to the burden caused by ill health [1]. Fever is normally a sign of a bacterial, viral or parasitic infection [2]. Besides fever, common symptoms that cut across several diseases include headache, general malaise, stomach pain and joint pain [3]. Various diseases cause these symptoms in Africa, most often respiratory infections, urinary tract infections (UTIs), malaria, dengue fever, typhus, typhoid fever, brucellosis and leptospirosis to name but a few [4–6]. Due to the large variety of fever causing infections with similar symptoms, febrile illnesses are notoriously difficult to diagnose clinically [7]. Hence, as a study in Tanzania found, there is a trend among health professionals to diagnose the cause of fever predominantly as malaria, urinary and upper respiratory tract infections, while leptospirosis, brucellosis and typhoid fever are rarely diagnosed. This reduces the number of times appropriate treatment is given thus eroding trust in health care [3].

In Morogoro, Tanzania, febrile diseases are common, and while malaria has substantially decreased, it is still endemic [5]. Additionally, livestock keepers are at an increased risk of contracting one of many febrile zoonotic diseases prevalent in the area [8]. Although professional health care remains the best option for diagnosis and treatment, the lack of proper diagnostic tools and a focus by clinicians on a narrow range of febrile diseases, contributes to misdiagnosis and inappropriate treatment. This, in turn, affects community trust in the health system [1, 3, 5, 6, 9]. To be able to tackle febrile illnesses and the morbidity and mortality they cause, it is also important to understand the treatment-seeking behavior of the affected population so that appropriate intervention strategies can be developed.

Whereas previous studies have noted various determinants of health-seeking behavior in Tanzania including finances and accessibility to a health facilities, many only capture the perspectives of those who are in the healthcare system, thus missing out on those who are treated with over-the-counter drugs and traditional methods [10]. Overall there is a paucity of studies that include socio-cultural and ecological factors [11–13]. The complexity of health-seeking behavior and access to health care has already been emphasized. In addition, many analyses have been criticized for their reductionistic approach which delineates individual actions from larger cultural and policy issues [12]. The decision making process determining the course of action undertaken is an intricate process affected by socio-cultural norms, values, quality of care, trust issues, economic considerations, and relational and structural factors [14–16]. The socio-ecological model is therefore useful for understanding human behavior by focusing on the multiple spheres of influence which can lead to the development of successful and sustainable solutions [17]. This model acknowledges that there are motivators and barriers for individual actions [18, 19]. Seeking health care is an iterative process determined by individual, financial, cultural, pragmatic and contextual considerations. The lay awareness, perceptions on diseases etiology, progression and severity of the illness and treatment play a big role in determining the actions taken when ill [20, 21]. Informal avenues of seeking health care such as traditional medicines and unprescribed medication from local shops are widely used in Africa since they are both affordable and accessible [4, 22].

Community based studies have been used to understand treatment-seeking actions regarding febrile illnesses in other settings [16, 22]. These qualitative studies were able to capture detailed and nuanced information at the household level. For instance, in one study on severe malaria in children in Tanzania, health-seeking behavior was found to be influenced by family relational dynamics, finances and structural factors [16]. Attitudes towards disease severity also determined the steps that patients took in seeking care for malaria in studies conducted in Kenya [22, 23]. Consequently, in order to enhance the control and timely management of febrile illnesses, a proper elucidation of facilitators and barriers towards proper health-seeking behavior is needed. This will lead to targeted health messages and broader policy decisions that increase accessibility to proper diagnostic and treatment facilities, thus curtailing the need to rely on sub-standard and inefficient options.

### Theoretical background: socio-ecological model

This study was guided by the socio-ecological model which was initially conceptualized by Urie Bronfenbrenner in the 1970s. The idea was to understand human development noting that individuals were influenced by various elements in their environment [24]. This concept was illustrated with concentric circles with the individual at the center surrounded by the various elements [25]. In this concept, the closest circle to the individual is the microsystem which consists of the individual’s immediate setting such as family, school and workplace. This is followed by the mesosystem which comprises the social system including peers and religious organizations. Lastly, there is the exosystem, which is the outer circle. This includes formal and informal structures including the neighborhood, social networks, the media and the government [18]. This model has been revised over the years and summarized to include individual, community (socio-cultural), institutional (organizational) and policy factors [17]. This model is adaptable and useful in understanding treatment-seeking behavior since it focuses on multiple levels of influence [18] as shown in Table 1.

**Table 1:** Socio-ecological determinants of treatment-seeking behavior

Levels of influence	Characteristics
Individual	Gender, age, economic status, level of education, knowledge, attitudes, personality, previous experience
Socio-cultural	Notions on syndrome causation and severity, family, social networks, peers, cultural background, gender roles, values, norms
Ecological	Weather, terrain, livelihood activities
Policy	Health system, laws, regulations, health policies, formal and informal health structures, partnerships

In the context of this study, this model is used to demonstrate that an individual’s treatment-seeking behavior is determined not only by personal characteristics such as gender, age and economic status, but by broader socio-cultural, ecological and policy factors [17]. A proper understanding of these levels of influence would lead to targeted solutions across each level that promote sustainable health-seeking behavior. Drawing from the pillars of the model, individual level influencers included knowledge, age and attitudes such as previous experience with a particular febrile illness. These determined a person’s treatment-seeking actions during subsequent illness episodes. At the socio-cultural level, perceived disease severity, beliefs on causation, family dynamics and social networks played a role in determining the actions people took. Ecological level influencers included weather, terrain and seasonality which influenced the organization of livelihood activities. Structural level factors included laws, and both informal and formal health structures. Accordingly, we examined the individual characteristics, social cultural factors and ecological and structural determinants influencing lay treatment-seeking actions among agro-pastoralists in the Kilombero district.

## Methods

### Study setting

This study was conducted over a period of 6 months between March and August 2019 in the Kilombero District, Morogoro region, in South Eastern Tanzania. The district has a population of 407,880 people [26]. The Kilombero district has two major hospitals, 3 health centers and 35 dispensaries. The predominant ethnic communities are the Wapogoro, Wandamba, Wabena and Wangoni, while the minor ones are the Wahehe, Wasukuma and Wamaasai. The Wasukuma and Wamaasai have moved into the district within the last two decades and keep large herds of livestock [24]. Rice, maize, cassava and millet are the main food crops grown and there are teak and sugar cane plantations. The study site was chosen because malaria is endemic in the area. Other febrile diseases including typhoid fever, respiratory and urinary tract infections, dengue fever, chikungunya virus, brucellosis and Rift valley fever are also prevalent [3].

Purposive sampling strategy was used to select three villages in the district. These villages were Signal, Sagamaganga and Lungongole. They were specifically selected due to the presence of agro- pastoralists who interact closely with both livestock and wildlife, a risk factor for fever-inducing zoonotic diseases. The agro-pastoralist communities in these villages also lived on the fringes of village settlements so they could easily access the communal grazing lands located next to the wildlife management area. This potentially increased their risk for zoonotic febrile illnesses due to close proximity to wildlife [3]. Access to health care was limited because of the remoteness of the settlements and poor access to roads especially during the rainy season between March and May when flooding is common.

### Data collection

Data were collected through in-depth interviews. Purposive sampling was used to identify participants who had experienced a febrile illness in their household, known locally as “*homa*”, within the 3 months preceding the study. These responses had been captured in a previous survey interview conducted by these same researchers on brucellosis awareness, risk perceptions and animal handling practices (Mburu et al. “Lay attitudes and misconceptions and their implications for the control of brucellosis in an agro-pastoral community in Kilombero district, Tanzania”, under review). Consequently, the researchers identified respondents from the survey who had responded affirmatively to having had a case of febrile illness in their household in the previous 3 months. A sample was obtained for in-depth interviews through the help of local village elders. Variations in age, gender, education level and religion were taken into consideration in order to gain diverse perspectives. All interviews were conducted in the National language of Kiswahili, while interview notes were taken by a trained research assistant. The interviews were conducted in convenient locations for the interviewees, mainly their homes. All the interviews were audio-recorded and each interview lasted between 45 min to 1 h. An interview guide was used to direct the discussions (Additional file 1) which comprised of questions on the respondents’ perceptions and experiences with febrile illnesses, as well as the actions taken and the reasoning behind them during suspected febrile illness episodes. Thirty-nine (28 male and 11 female) interviews were conducted and no new participants were recruited after realizing that saturation had been reached.

### Data analysis

All in-depth interviews were recorded and notes taken. The audio files were transcribed and translated into English by one of the research assistants, who is fluent in Kiswahili and English. To check the accuracy of the original transcription and translation process, back translation was conducted by a second translator assisted by ME who is a native Kiswahili speaker. The transcripts were reviewed by CM, SB and ME by comparing the scripts to the audio files. The field notes were also used as a further source of verification before validation. The data was entered into the NVIVO 10 software (QSR International, Australia) for the development of codes. This allowed the researchers to manage the data, organize ideas, define themes and derive conclusions from this study in an iterative and collaborative manner [27, 28]. CM developed the initial codes which were validated by SB and ME. The coding process involved categorizing the data to identify the ideas that related to each other while noting the similarities and differences among them. These codes were thereafter classified into themes in order to identify meanings and patterns in line with the study objectives and draw the relevant conclusions. Translated verbatim quotes have been used to illustrate the key points of this study.

## Results

### Respondents’ socio-demographic characteristics

A total of 39 (28 male and 11 female) informants participated in this study as shown in Table 2. Twenty-one were from Sagamaganga village while 12 and six were from Lungongole and Signal villages respectively. All were agro-pastoralists. Slightly more than half (54%), did not have formal education and most (90%) were married and belonged to the Wasukuma tribe. Close to half (49%) of the participants were above 42 years of age. 59% of the informants practiced the local religion.

**Table 2:** A Summary of the socio-demographic characteristics of the participants

Demographic characteristics		In-depth interview participants (No.)
**Village of origin**	Sagamaganga	21
	Lungongole	12
	Signal	6
**Sex**	Male	28
	Female	11
**Age**	18–25 years	5
	26–33 years	9
	34–41 years	6
	42–49 years	11
	> 50 years	8
**Education**	None	21
	Primary	11
	Secondary	4
	Tertiary	3
**Religion**	Local religion	23
	Christian	14
	Muslim	2
**Ethnicity**	Wasukuma	34
	Others	5
**Marital status**	Married	35
	Single	3
	Widowed	1

### Determinants of actions taken during a suspected febrile illness episode within the socio-ecological model

In this study, by using the socio-ecological model, various determinants were found to influence treatment-seeking behavior during self-reported febrile illness episodes as presented in Table 3 below. These are discussed in more detail in the subsequent sections.

**Table 3:** Summary of the determinants of agro-pastoralists’ treatment-seeking behavior during a suspected febrile illness episode using the socio-ecological model

Level of influence	Determinant	Illustration
**Individual**	Age	Children below 3 years of age were promptly taken to a health facility for fear of severe illness progression.
	Expendable income	Families with a low level of income were less likely to visit a health facility out of fear that they would not be able to afford the high costs associated with formal treatment.
	Personal history	Individuals took note of previously used drugs and purchased them from local shops and pharmacies anytime they had similar symptoms.
**Socio-cultural**	Community perceptions on febrile illness	Most febrile illness were perceived as malaria so antimalarials were purchased for treatment.
		Distinction was made between common and severe febrile illness based on perceived severity of the symptoms.
		Only malaria, typhoid and urinary tract infections were mentioned as causes of febrile symptoms.
		Malaria was associated with fever, chills and joint pains.
		UTIs were perceived to manifest through severe headaches and backache.
		The main typhoid symptoms were considered to be stomachache and constipation.
	Attitudes on formal and informal treatment	Recurrent severe febrile symptoms were associated with witchcraft. As a result, traditional healers were consulted.
		Informal drug sellers were considered to be knowledgeable on the cause of diseases and thus able to prescribe the right treatment.
		Clinicians were perceived as being able to conduct accurate diagnosis. For this reason, their help was normally sought once self-treatment failed.
**Ecological**	Seasonal livelihood activities	People were unwilling to leave their farming or livestock keeping duties to attend a health facility. Therefore, they preferred purchasing drugs over the counter.
		Herders were less likely to visit a health facility as they looked after livestock in remote areas with no one to be left in charge of the animals.
	Weather	Flooding for part of the year impeded transportation to a health facility.
**Policy/structural**	Accessibility to health services	Local shops selling medicine were more accessible than health facilities.
		Most of the tests were available in major hospitals located in towns far from the villages.
		Private facilities were closer to the villages and tested patients for malaria, typhoid and urinary tract infections.

### Individual level factors

Individual level factors including age, expendable income and personal history played a significant role in influencing the choice of action. Most of the respondents observed that buying over-the-counter medication was their first course of action. However, this was not the case when the illness affected the young or old, who were considered most at risk for severe febrile disease and were thus promptly taken to a formal health facility. Another motivator for the age-determined preferential treatment was that the old and young were treated at no cost in public facilities. The following excerpts demonstrate this:

*“For adults, we purchase over-the-counter drugs and only go to a health facility if very ill. But for little children and older people we take them to the hospital immediately. This is because the illness can progress dangerously fast in their case”.*

*Male (20-30 years), IDI Kilombero.*

*“We don’t buy drugs for little children since they are not able to fully express themselves and so we cannot tell if they are seriously ill or not”.*

*Female, (40-50 years), IDI Kilombero.*

>Available household finances also influenced what mode of treatment was chosen based on appropriateness and affordability. Self-treatment was preferred as it was a cheaper option. Seeking formal healthcare entailed not only the direct costs of treatment but also indirect costs such as lost productivity, as well as transport and meal costs for both the patient and accompanying relative. The quote below exemplifies this:

*“If I do not have enough money, I buy over-the-counter medication when sick. It is expensive to go to a health facility because I not only pay for tests but for transportation and food as well”.*

*Male (50-60 years old), IDI Kilombero.*

If subsequent action was required, however, participants noted that they borrowed money from neighbors and friends to enable them visit a health facility. Different options even at this stage were chosen based on economic ability or time of the year. Those with more money sought care in the private facilities, which were more expensive, to avoid the long queues in public facilities. If an illness occurred soon after a harvest when families had more income from sale of produce, then the sick could go promptly to a health facility. An individual’s personal history with febrile illnesses was used to determine what options to explore when that person got sick. Drug packaging from medicines taken previously was stored carefully in case of similar symptoms returning in the future, so the same drugs could be purchased and consumed. The perception was that the current illness could be cured using the same medication, thus avoiding a hospital visit. The statements below elucidate this:

*“Once we know the treatment for a disease that we have had in the past, we simply go and buy the same medication if such symptoms recur”.*

*Female (40-50 years old), IDI Kilombero*

*“We often become familiar with the symptoms and treatment administered. So, we note the drug or keep the packet in a safe place so that next time we can recall the medication and go purchase it over the counter”.*

*Male (50-60 years old), IDI Kilombero*

### Socio cultural level factors

The socio-cultural factors that determined people’s treatment-seeking behavior were their worldview on febrile illnesses and their attitudes on formal and informal treatment options. According to local perceptions a febrile illness had dual causes: naturalistic and personalistic. In this community, fever causing illnesses were attributed to natural causes including mosquitoes, poor sanitation and changes in weather. However, in cases where there was a long duration of illness in spite of medical treatment, the illness was attributed to personalistic causes, mainly sorcery and witchcraft. This was especially the case if the patient exhibited what were considered unusual signs, such as mental health issues. Consequently, self-treatment was the first course of action followed by hospital treatment, and lastly traditional healers in case of non-recovery. Malaria was perceived as the main cause of febrile symptoms and considered easily treatable through antimalarials. The findings of this study show, that a febrile illness (*homa* in Swahili) was considered one which caused a headache, body aches, general malaise, joint pains, chills and general discomfort. The following two citations exemplify this: *“You say you have a febrile illness when you have body aches, headache, general malaise, tiredness, headache and joint pains.”* (Male, 45 years). A 21-year-old female respondent observed that *“fever, headache, tummy ache, diarrhea, feeling very tired and pain all over the body are the signs of a febrile illness”.* These febrile symptoms were considered to be a sign of malaria, typhoid or a urinary tract infection. However, malaria was the most commonly cited cause of febrile illness and thus taking paracetamol and antimalarials was the primary course of action as demonstrated in the quotes below:

*“When the chills start then I know it is malaria and I go and purchase malaria drugs”.*

*Male (40-50 years old), IDI Kilombero*

*“When I have these symptoms including a headache, I will first take paracetamol for immediate relief and then buy and consume malaria medication”.*

*Female (50-60 years old), IDI Kilombero*

The lay people differentiated between what they considered an ordinary febrile illness *(homa ya kawaida in Kiswahili)* and a severe one *(homa kali in Swahili).* Ordinary illness was one where in spite of the illness, the patient was able to continue with most of their daily chores, was not bedridden and could eat, and this was often attributed to malaria. Severe illness was one where the patient was incapacitated, was unable to eat, walk and perform their daily activities. At this point a visit to a health facility was considered necessary to identify the cause of ill health. These perspectives are illustrated by this excerpt:

*“If someone is sick with chills and joint pains but can still walk and talk then we know it is a regular illness (homa ya kawaida) and so we buy them malaria drugs. However, if someone can’t eat, sit, is in pain and in bed all the time then we know the person is seriously sick (homa kali) and so we quickly take then to the hospital”.*

*Male (40-50 years old), IDI Kilombero*

In addition, the community concluded that they were suffering from either malaria, typhoid or a UTI based on observable signs. In most of the cases, different sets of symptoms were associated with different diseases. Fever, chills and joint pains were invariably attributed to malaria, a severe headache and backache to urinary tract infections, and stomach pain and constipation to typhoid. This consequently led them to either purchase specific medicine in the shops or to go to a health facility for treatment. This self-diagnosis is elucidated through the quote below:

*“I can tell what I am suffering from. When it is malaria, I get chills and joint pains, a severe headache when it is a urinary tract infection and for typhoid, I get a stomachache and constipation”.*

*Male (20-30 years old), IDI Kilombero.*

Whenever a febrile illness persisted despite the use of formal healthcare facilities, a traditional healer was consulted. This was because the illness was attributed to supernatural causes such as witchcraft or sorcery. However, if the traditional methods also failed, then the patient went back to the formal healthcare system for treatment. This utilization of multiple, concurrent options is elaborated through the statement below:

*“We go to the traditional healers when we have a persistent febrile illness. They can determine if one has been bewitched or if God has sent the disease to you. If that is the case even if you keep going to hospitals for tests the doctors cannot identify the disease. And usually, you go to a healer after trying hospital medicine in vain. They can tell if you have been bewitched and then prescribe the right treatment”.*

*Female (30-40 years old), IDI Kilombero*

Formal and informal service providers were considered as complementary and were utilized on different occasions depending on the perceived cause and severity of the illness. Treatment-seeking was an iterative process with people navigating across a broad spectrum of care providers. Clinicians were highly regarded because they could conduct tests and determine the exact cause of illness. Drug sellers in local shops were considered experts in the prescription of the right and appropriate medication. Traditional healers fell in the other spectrum of expertise because they came into play only after the first two options failed to work. The rationale being that supernatural forces could be the cause of the illness which could not be addressed using conventional drugs. These perspectives are captured in the statements below:

*“Those selling medicines are experts and so we trust them when they recommend treatment. However, if we don’t feel better then we go to a hospital where they can conduct tests. Female (40-50 years old), IDI Kilombero*

*“Doctors are good but sometimes they also fail so we change strategy. But we don’t tell the doctor that we went to a traditional healer because they don’t like that. And if the healer fails to cure my illness, then I go back to the doctor and lie that I was looking for money for health care or I was using over-the-counter medication. But if I get well then I might disclose to the doctor that the traditional healer cured my ailment”. Male (40-50 years), IDI Kilombero*

### Ecological factors

In the study area, two key ecological factors were found to play a critical role in determining what people did when they observed any febrile symptoms. These were seasonality and weather, which determined how livelihood activities were structured and the individual roles that were expected of family members. These influenced the decisions on when and where to seek treatment. The participants in this study engaged in seasonal work especially on the farms, and thus they were very busy during the planting, weeding and harvesting season between February and July each year. The less busy season for the farmers was after the harvest in August–November before they started to prepare their fields again for the next planting season. In the busy months many opted for over-the-counter drugs, while in the less busy season they could go to the public health facilities as they had time to queue and money to spend after selling their produce.

*“It depends on how busy I am. If it is during the planting season, I buy over-the-counter medicine and take it, but like now, when I am not very busy, I will go to the hospital because I have time to wait in line”.*

*Male (30-40 years old), IDI Kilombero*

Secondly, cattle were usually taken approximately 8–12 km away from the homes to graze in the communal grazing lands where the herders camped for up to 9 months each year between June and February of the following year. These grazing areas are in isolated places where healthcare access is limited. One or two young men from each family would go to look after livestock carrying paracetamol and antimalarials to take when they got symptoms suggestive of a febrile illness. These were preparations to avoid the inconvenience of having to return home to seek treatment. As one herder observed:

*“For us herdsmen since we live away from the village and health facilities, we purchase the drugs, take them with us and store them well and if one is sick then he takes them. It is hard to walk all the way back to the village when you are sick and also there may be no one else available to look after the herd”. Male (20-30 years old), IDI Kilombero.*

The weather was another important factor, because the study area is prone to flooding for about three to 4 months each year. When it floods, movement is impaired as the roads are impassable. This caused most of the people to purchase drugs or use herbs due to the limitations of travel.

*“It is very hard for us during the rainy season here. Roads are flooded and motor bikes which we use for transport cannot get to our homes. It is especially hard for us women and so we use herbs”.*

*Female (40-50 years old), IDI Kilombero*

### Policy factors

Accessibility to health facilities and other formal and informal health services was a key factor in influencing treatment-seeking behavior. Since the agro-pastoral communities lived on the fringes of the settlements, accessibility to health facilities was seriously impeded. Although health centers were located in the villages, these facilities often lacked drugs and diagnostic capacity and patients had to purchase over-the-counter medication or visit a bigger health facility approximately 8-15 km away. *“Often, the health centers lack drugs and so one has to go buy medicine outside the hospital which is more expensive.*

*Male (20–30 years old), IDI Kilombero.*

The option to visit the bigger facility was used as a last resort due to financial constraints and the long queues in the hospital. Local shops selling medicines and private treatment centers, on the other hand, were located in the villages and also tested for malaria, typhoid and urinary tract infections and thus were widely popular and used due to ease of access.

*“There are always long queues in our health facilities which make us prefer purchasing medicine than queuing all day. We have a private laboratory here in the village which tests for malaria, typhoid and UTI and then one goes to buy medicine in a shop depending on the test results”.*

*Male (40-50 years old), IDI Kilombero.*

## Discussion

The aim of this study was to explore the factors that determined the steps taken by community members during a febrile illness using the socio-ecological model. The findings of this study show that treatment-seeking behavior during a febrile illness episode is an iterative process influenced by various factors, as elaborated within the socio-ecological model. At the individual level, age was a significant determinant, with the very young and old prioritized for immediate, professional medical attention. This was attributed to their perceived vulnerability to severe disease and because they were often treated at no cost, which reduced the economic burden on families. This demonstrates that the lay people perceived febrile illnesses as having the potential to be severe among the weak and vulnerable. Similarly, other studies conducted in Tanzania, demonstrated that children, especially those below five, were prioritized for biomedical attention through anti-malarials and hospital care which was related to the sensitization of caregivers on the dangers of febrile illnesses in under-five year old children [12, 14, 29].

A second determinant identified was limited expendable income, whereby most people purchased over-the-counter drugs due to lack of finances to pay for services at the health facilities. As a result, many opted to self-medicate and only went to a health facility in the event that they failed to recover. This inevitably would cause delayed treatment and increase the likelihood of chronic symptoms developing, leading to more suffering. Inadequate financial capacity has been implicated in the delay in seeking prompt medical attention in other studies. Others have identified the preference for the purchase of over-the-counter drugs because of the low cost involved [30, 31]. In a study in rural Mali, economic constraint was the main barrier to participants seeking treatment from a formal healthcare facility [32]. Another important factor in this study was the previous history of a febrile patient. The medications used in the past were noted and used in the event of similar symptoms in the future. This was used to cut costs and to avoid having to visit a clinician, which was deemed expensive and time consuming. There is therefore the risk of incorrect treatment as a result of this behavior. In a previous study in Tanzania, mothers, based on their previous experience, administered malaria medicine to their feverish children based on the belief that these same drugs would be provided in a health facility anyway [12].

At the socio-cultural level community perceptions on febrile diseases were found to play a remarkable role in determining the kind of treatment sought. The study found that a febrile short-term illness was attributed to natural causes while chronic febrile signs were perceived to be the result of supernatural causes. The key symptoms associated with a febrile illness in this study were headache, fever, chills, joint pains, backache and stomach pain which is congruent with a febrile disease [4]. The only diseases associated with febrile signs by the respondents were malaria, typhoid and urinary tract infections (UTIs). Malaria, in this study, was the most commonly perceived cause of febrile symptoms, thus indicating low knowledge on the broad spectrum of febrile diseases and the need for increased sensitization and education of the community. Other studies in Kenya, Tanzania and Ethiopia had similar findings where febrile symptoms were attributed to malaria leading to the purchase and use of over-the-counter malaria medication. In this study also, specific signs were associated with either malaria, typhoid or urinary tract infections. For example, fever and chills were attributed to malaria and stomach pain to typhoid. This practice of avoiding proper diagnosis before starting treatment, can lead to mal-treatment and delays in accurate diagnosis which can impede a timely and full recovery.

In a similar study conducted in the Kilosa district neighboring the Kilombero district in Tanzania, patients presenting at hospital with febrile symptoms were diagnosed with malaria, typhoid fever, pneumonia and urinary tract infections, while no cases of brucellosis, leptospirosis and typhoid were clinically diagnosed [3]. This under-diagnosis and lack of treatment of other febrile diseases may lead to chronicity and consultation of traditional healers, further exacerbating the condition. Febrile illnesses are due to a variety of causes and were repeatedly mis-attributed to malaria, typhoid and UTIs. Conversely, a study conducted in Ifakara and Dar es Salaam in Tanzania found that viral infections were the major cause of febrile disease among children and not malaria [1]. In the Kilosa District of Tanzania, febrile cases in children were attributed to malaria (23%), leptospirosis (13%), brucellosis (10%), typhoid (10%) and urinary tract infections (19%) with co-infections in 39.5% of the cases demonstrating the variability of fever causing infections [3].

The lay people differentiated between ordinary and severe disease based on e how ill the person appeared. When fully incapacitated the illness was considered severe and ordinary when normal daily activities could be performed. Although some diseases such as malaria can cause severe illness, other diseases fail to cause severe illness at first but become chronic such as brucellosis. Therefore, this perception of ordinary vs. severe illness can be misleading, invariably leading to delay in obtaining the right care. Others have observed that the biomedical model of defining disease and illness including severity is insufficient because of its reductionist nature and does not cater for the broad spectrum of lay people’s perceptions and experiences of ill health. Ill health is experienced to varying degrees by individuals and sensitization efforts need to take these varying perceptions into consideration. How people label and categorize a disease determines the steps they take in curing it, especially when deciding whether traditional or conventional treatment will be sought [33, 34]. Community based studies of health-seeking behavior show that perceived severity of a disease influenced the subsequent actions taken, with poorer people delaying treatment [12, 20, 23]. In one study in Uganda, it was found that seeking care for a child who had a fever was dependent on perceived severity of the illness with some illnesses being regarded as requiring traditional therapy [33]. In another study, when the cause of disease was attributed to supernatural forces, the people were hesitant to take any preventive actions because they felt that they had no control on whether they suffered from the illness or not [35].

Formal and informal healthcare providers were valued for their variable expertise and ability to address a fever causing illness. Clinicians were regarded highly because they could perform tests and ascertain the cause of the illness. Pharmaceutical drugs sellers were perceived as capable of recommending the right treatment. Traditional healers too were considered competent in treating recurrent febrile illness. On their part, the traditional healers used charms and herbs to treat the illnesses and more than one healer could be used in an attempt to find a cure. If that failed, patients reentered the hospital system. These pathways were used based on the perceived etiology of the illness. This shows that local perceptions on the abilities of healthcare providers play a big role in determining their treatment-seeking behavior. Our findings are similar to those of a community-based study in Tanzania which found that traditional healers were sought once there was a persistent fever after hospital treatment and many did not seek further immediate medical attention [36]. Such findings contradict the findings of a study conducted in Tanzania, which found that local healers were not utilized as much as expected, because people used over-the-counter pharmaceuticals, which were deemed a lot more effective [37]. This behavior shows that there are many opportunities for misdiagnosis and wrong treatment, which could eventually lead to debilitating chronic disease [38]. Critically, when chronic symptoms develop, they may be further attributed to witchcraft because of the length of time needed to treat them.

The ecological determinants identified in this study were weather and seasonal livelihood patterns. These are important because they served as a barrier to proper medical attention. Agro-pastoralists were hesitant to seek care in a health facility during certain periods of the year as it would derail them from their work. This was due to the long time it took to go to a health center and the long queues in the facilities which served as a hinderance. Corroborating these findings, others have found that vulnerability to severe disease was enhanced during the rainy season (January to April) in the Kilombero district, when local people were busy planting and weeding [39]. During this time of the year, food was in short supply. Coupled with flooding, it was difficult for people to access the correct treatment due to limited household finances. The implication is that people’s livelihood strategies impact how people deal with disease and their health-seeking practices. Improving livelihood and income through profitable animal husbandry and farming would go a long way in ensuring better compliance with formal healthcare seeking. People would have lighter workloads and a better income [39]. This is similar to what was observed in a study in Uganda where care seeking for a child was found to be affected by involvement in household responsibilities by the caregivers [40]. Another study conducted in Palestine found that women’s treatment-seeking behavior was negatively affected by their role in child care and household duties [35].

Accessibility to health services is a major structural determinant of health-seeking behavior. In line with this theoretical postulate, this study found that over-the-counter drugs were preferred because of ease of access and low cost, which is similar to other studies [12, 22]. In this area, government health centers and hospitals were less accessible compared to local shops and private health facilities. Shops where the informants could purchase over-the-counter medication were located within the villages and were open for business for much longer. This caused people to utilize the latter more. Public facilities also often had very long queues which caused many to prefer over-the-counter medication. Therefore, provision of timely public and affordable health facilities would encourage patients to seek help in formal facilities.

In one study, a major contributor for the self-treatment of febrile conditions in Tanzania was the ease of access of private laboratory facilities and medicine shops than formal health facilities with people visiting a public health facility when the symptoms worsened [41]. Concurringly, in his study, access to a health facility was a far more significant determinant of health-seeking behavior than local disease perceptions on etiology in rural Tanzania [16]. As other studies have demonstrated, over-the-counter drugs from local shops are preferred as initial treatment because they are easy to access compared to health facilities [35]. Since most rural people use these informal services, their regulation and standardization would enhance the provision of appropriate and correct diagnosis and treatment and thus contribute to better health outcomes for the local people. This study demonstrates the dynamic and iterative nature of treatment-seeking behavior in a rural community during febrile illness episodes. It is influenced by various factors which enable or constrain individual actions. Specific mitigation efforts would go a long way in ensuring proper healthcare is sought in a timely manner, reduce reliance on self-treatment and the seeking of help from unqualified individuals.

### Study limitations

This study was dependent on the ability of participants to recall their behavior during what they considered a febrile illness episode in the past, thus introducing recall bias. However, this study depended on recall of a situation within the previous 3 months and notably febrile illnesses are common in the area [3]. This means that the participants have to deal with a febrile illness in the household on a fairly regular basis, thus enhancing the validity of the data. The second limitation is that the study relied on a self-determined febrile illness and symptoms and no clinical data or tests were conducted to verify the symptoms and obtain a clinical diagnosis. Nevertheless, the symptoms identified by the participants closely relate to the biomedical classification of a febrile illness.

## Conclusions

This study found that factors at various levels of the socioecological framework influenced treatment-seeking behavior. Therefore, multi-disciplinary strategies across all the levels of influence are needed in order to improve health outcomes. Messaging on the diversity of febrile illnesses is key to help the local community understand the nature and diversity of febrile diseases. This is especially crucial since zoonotic diseases such as brucellosis present as a febrile illness too and affect especially the livestock keepers. Training livestock keepers to engage in profitable agricultural activities would also enhance family income and consequently their ability to seek formal health services. There is a need too for facilitating access to prompt treatment through improving infrastructure and the diagnostic capacity in the dispensaries and health centers. This could reduce reliance on untrained individuals offering treatment for febrile illnesses.

## Supplementary Information

**Additional file 1.** In depth Interview Guide.

## Data Availability

All the data used in this study is available through the corresponding author.
