# Supplementary material for: Determinants of treatment-seeking behavior during self-reported febrile illness episodes using the socio-ecological model in Kilombero District, Tanzania
Source: BMC Public Health. 2021 Jun 5;21:1075. doi: 10.1186/s12889-021-11027-w (PMC8180143; doi:10.1186/s12889-021-11027-w)
Supplement: Supplementary file 1 — Additional file 1. In depth Interview Guide. [file 12889_2021_11027_MOESM1_ESM.pdf]

Appendix 1: In depth Interview Guide

Introduction

My name is Caroline M Mburu. I am a PhD student at the University of Nairobi in Kenya. I am conducting this research to understand the views of livestock keepers related to brucellosis in livestock and in humans. This research is for my PHD degree at the University of Nairobi in Kenya. I am especially interested in the views of both men and women on animal husbandry practices, consumption habits and health seeking behavior. Everything that you share with me today will be used for the sole purpose of this research only. Your name will not be used and you have already consented to this interview using the consent form. Do you have any questions before we begin?

Background Information

No of interview:

Age:

Sex:

Marital Status:

Level of education:

Religion:

Introduction:

1.Please tell me the benefits of livestock

Probe: Cattle, goats, sheep

2.Kindly tell me how you acquire new stock

Probe: From where, transportation, what do you look for

3.Name the common livestock diseases in this region

Probe: Local names, Signs of each disease, Severity of diseases, Seasonality of diseases

2. Human- Animal interface dynamics

4.What do you know about zoonotic diseases?

Probe: Which ones, Risk factors

5.Please tell me about the gender roles in this household

Probe: Milking, herding, assisting in births and wearing of gloves, Residing with livestock, curing hides

6.What do you think about the consumption of raw milk/raw blood/raw meat?

Probe: Reasons why, when, who consumes it, boiling milk perceptions

7.What typically happens when an animal dies?

Probe: Consumption, how meat is considered safe/unsafe and any actions taken

8.Which diseases cause abortions in livestock*? (NB:B abortions between 5th-7th month*).

Probe: Stage at which abortions occur, one off or repeatedly, Why they occur, Actions taken, Abortion storms, Disposal of aborted fetuses

9.What do you think is the causes(s) of retained placenta in your livestock?

Probe: How is this addressed

10.What leads to infertility in your livestock?

Probe: Why, what is done, benefits or losses incurred, males and females

11.Please tell me about still births in your livestock?

Probe: Why, Actions taken

12.What do you think about the birth of weak calves or kids in your herd?

Probe: Why, Any actions

13.What causes reduced milk yield in cattle?

Probe: How do you determine reduced milk yield? How is that interpreted?

14.What is your opinion about about swollen/enlarged/inflamed joints in a cow?

Probe: Causes, Actions taken

Environmental interactions

15.Please tell me what typically happens when cattle are being moved from one place to another?

Probe: Migration, Who, Why, Where

16.What do you think about the interaction of livestock with wild animals?

Probe: Which wild animals? Where? Why? Movement of wild animals, Knowledge of potential risks? Gender and Age of people involved

Treatment pathways for febrile illnesses

17.Kindly name the common human diseases in this locality that cause fever.

Probe: Local names for fever and meaning, undulant fever, other common symptoms, causes, progression, degrees of severity

18.What kinds of treatment are sought for febrile illnesses?

Probe: How soon, Progression in seeking care, Differences between males and females, Children and adults, Utilization of health care facilities, Over the counter drugs, testing, alternative treatment sources

19.Please tell me about the challenges you encounter in accessing treatment for febrile illnesses

Probe: Duration of illness, Cost, Accessibility of treatment, Preferred treatment, gender differences

20.Kindly tell me about the difficulties you face after the treatment for a febrile illness

Probe: Any recurring symptoms, Actions taken

Closing questions

21.What are the major challenges you face as an agro pastoralists related to livestock diseases?

Probe: Zoonotic diseases

22.What do you think needs to be done to mitigate these challenges?

Probe: Access to veterinary services, treatment of livestock, awareness and education to local community
